# Child Welfare Involvement and Health Outcomes in Infants With Prenatal Substance Exposure

**DOI:** 10.1001/jamahealthforum.2026.1302

**Published:** 2026-06-05

**Authors:** Wan-Ting Chen, Christian M. Connell, Sarah A. Font

**Affiliations:** 1 Human Development & Family Studies, College of Health and Human Development, The Pennsylvania State University, State College; 2 Child Maltreatment Solutions Network, Social Science Research Institute, The Pennsylvania State University, State College; 3 Brown School of Social Work, Washington University in St Louis, Missouri

## Abstract

**Importance:**

The child welfare system (CWS) is often contacted when a child is born with prenatal substance exposure, but there is limited evidence on the beneficial and iatrogenic effects of CWS involvement.

**Objective:**

To examine whether involvement with the CWS at birth is associated with injuries and moderate- to high-complexity emergency department (ED) use in the first year of life among infants identified with prenatal substance exposure.

**Design, Setting, and Participants:**

Retrospective cohort study using probabilistically linked administrative data of Pennsylvania statewide child welfare administrative records linked to Medicaid claims. Births from January 1, 2015, to December 31, 2018, were followed up for 12 months. Data were analyzed from October 9, 2025, to March 21, 2026. A sibling fixed-effects design compared infants born to the same mother who differed in their CWS involvement status. The cohort included Medicaid-covered infants identified with prenatal substance exposure who were named in confirmed child welfare cases at birth involving alleged maltreatment or a sibling and met continuous Medicaid enrollment and successful linkage criteria. The discordant sibling sample included infants born to mothers with at least 2 infants who differed in involvement status.

**Exposure:**

Involvement with the CWS in the birth month.

**Main Outcomes and Measures:**

Primary outcomes included any injury, serious injury (Abbreviated Injury Scale score ≥3 or CWS-documented fatalities/near fatalities), and moderate- to high-complexity ED visits (*Current Procedural Terminology* evaluation and management codes 99283-99285; critical care codes 99291-99292), including repeat visits. Secondary outcomes included well-child visits, low-resource-intensity ED visits, and maternal postpartum care.

**Results:**

In this study in 5858 of infants (3001 male and 2839 female) identified with prenatal substance exposure, by age 1 year, 14.5% had an injury-related claim, 33.8% had a moderate- to high-complexity ED visit, and 14.3% had a repeat ED visit. In sibling fixed-effects models, involvement with the CWS at birth was associated with a 20% relative reduction in moderate- to high-complexity ED visits (−7.3 percentage points [pp]; 95% CI, −10.8 to −3.9 pp) and a 24% relative reduction in repeat ED visits (−3.7 pp; 95% CI, −6.3 to −1.2 pp). Conversely, on-time well-child visits were 15% lower (−7.8 pp; 95% CI, −11.7 to −4.0 pp) for CWS-involved siblings.

**Conclusions and Relevance:**

Results of this study suggest that child welfare involvement is associated with reduced health care use among infants with prenatal substance exposure, especially moderate- to high-complexity ED use.

## Key Points

**Question:**

Is child welfare system involvement at birth associated with health and safety outcomes in the first year of life for infants with prenatal substance exposure?

**Findings:**

In this cohort study in 5858 Medicaid-covered infants identified with prenatal substance exposure who were named in confirmed child welfare cases involving alleged maltreatment at birth or a sibling, involvement at birth was associated with a 24% relative reduction in repeat moderate- to high-complexity emergency department visits in the first year of life; findings for injuries were not significant. On-time well-child visits showed a 15% relative reduction.

**Meaning:**

Results of this study suggest that early child welfare involvement is associated with a reduction in moderate- to high-complexity emergency department use for infants with prenatal substance exposure.

## Teaser

This cohort study reports on whether child welfare system involvement in infants prenatally exposed to substances is associated with health and safety outcomes in the first year of life.

## Introduction

Parental substance use is a major factor in child welfare system (CWS) involvement and infant harm.^1,2^ Infants with prenatal substance exposure (PSE) face elevated risks of injury, neglect, and death.^3,4^ Drug-related infant fatalities in the US more than doubled between 2018 and 2022, with most deaths occurring postneonatally (28-364 days).^5^ Under the Child Abuse Prevention and Treatment Act (CAPTA), as amended by the Comprehensive Addiction and Recovery Act of 2016,^6^ medical practitioners must notify state authorities of substance-affected infants to ensure service linkage through Plans of Safe Care. While not necessarily filed as formal maltreatment reports, the reports with prenatal substance exposure (PSE) are managed by child welfare agencies and often handled by the same caseworkers. Yet, due to inconsistent toxicology testing,^7^ unreliable screening,^8^ and varied definitions of substance-affected, many INFANTS WITH PSE are never diagnosed or subject to notification.^9,10,11^ Evidence from California and Washington suggests that 40% to 60% of diagnosed infants with PSE do not receive a formal report or notification at birth,^12,13^ although rates vary across jurisdictions. Practitioner concerns about the consequences of reporting (eg, stigma, reduced treatment engagement, child removal) may also play a role.^14^ Still, efforts to avoid early CWS involvement may only delay later involvement, given the association between parental substance use, maltreatment, and CWS involvement.^10,15^

Infants with PSE are vulnerable due to both biological effects of substances and environmental adversity. Substance use during pregnancy can disrupt fetal neurodevelopment and lead to neonatal withdrawal, impaired regulation, and other complications that increase the need for medical care in infancy.^16,17,18^ Neonatal abstinence syndrome is associated with higher rates of emergency department (ED) visits in the first year of life than matched peers without neonatal abstinence syndrome.^17^ Beyond the direct effects of exposure, ongoing maternal substance use may increase the risk of injury or adverse health events, particularly when mothers are primary caregivers. CWS involvement can increase access to maternal substance use disorder (SUD) treatment,^2^ and addressing SUD may improve supervision, responsiveness, and engagement with preventive care, thereby reducing injury risk.^19,20,21^ Substance-specific hazards, such as indoor exposure to cannabis smoke,^22,23^ may also be reduced with CWS intervention and monitoring. Alternatively, many CWS cases do not result in any service provision, so involvement may have little impact on infant outcomes. CWS involvement could also increase harm if mothers avoid treatment or other supports out of fear of custody loss or perceived stigma.^24,25^

Maternal substance use often occurs within a broader context of mental health challenges, trauma histories, violence, unstable housing and relationships, poverty, and limited access to supportive services.^26^ These factors may increase infants’ exposure to unsafe or unhealthy environments and elevate the risk of injury and ED use.^27,28,29^ They may also compromise mothers’ access to adequate preventive care for their infants, although the evidence is inconclusive on the role of preventive care in reducing ED use.^30^ Prior research shows that formal CWS involvement, especially foster care, increases preventive care use among young children.^30,31,32^ There is an ongoing debate about the potential harms and benefits of CWS involvement at birth for infants with PSE. CWS involvement may not occur if a newborn is not subject to a CAPTA notification or the notification is unaddressed or diverted to voluntary services without investigation.^33^ A recent study from Connecticut, where notifications largely became deidentified and received no CWS response, found that diversion reduced CWS reports and foster care entries.^34^ Yet, the implications for child health and safety remain largely unexplored.

This study uses linked CWS and Medicaid claims data to assess whether CWS involvement at birth is associated with infants with PSE. To reduce selection bias, we used a sibling fixed-effects design using discordant siblings (families where at least 1 child was involved with CWS at birth and at least 1 was not) to examine associations between CWS involvement and ED visits and injuries in the first year of life. We also assessed health care avoidance (mothers limiting contact with health care practitioners following CWS involvement) as an alternative explanation for our findings.

## Methods

### Study Design and Setting

We conducted a cohort study using data of Pennsylvania statewide child welfare administrative records linked to Medicaid claims. Under Pennsylvania Act 146 of 2006,^35^ health care practitioners must notify the state if an infant is affected by PSE, meaning they exhibit observable symptoms of exposure, such as withdrawal. Other concerns about parental substance use upon delivery (including concerns about substance-exposed newborns who did not display physical symptoms) may be reported to the state hotline, but this is not mandatory. Neither concerns about parental substance use nor notification of a substance-affected infant constitutes a maltreatment report. Instead, such concerns are assigned to Pennsylvania’s alternative response system (general protective services) and can lead to outcomes ranging from no intervention to referral to voluntary services, monitored in-home services, or placement. Due to Pennsylvania’s strict expungement laws, CWS reports where no allegations are validated are fully deleted within 1 year and 3 months. Thus, our data comprise only alternative response cases and reports with at least 1 confirmed allegation: the subset of cases where CWS identifies concerns warranting possible intervention. The study was approved by the Pennsylvania State University Institutional Review Board with a waiver of informed consent due to analysis of secondary data. Reporting followed the Strengthening the Reporting of Observational Studies in Epidemiology (STROBE) reporting guideline.

### Data Sources

We used Pennsylvania CWS administrative records probabilistically linked to Medicaid claims for children and biological mothers. Births from January 1, 2015, to December 31, 2018, were followed up for 12 months. Data were analyzed from October 9, 2025, to March 21, 2026. Data linkage procedures followed previously validated matching methods.^2^ CWS records include children who are the subject of an allegation (including parental substance use or infants with PSE concerns) as well as their siblings who may not be the subject of any allegations. Our state research agreement does not include Medicaid-covered births for children who were never named in an allegation of maltreatment on a CWS case.

### Study Population

The study population was children from Medicaid-covered births in Pennsylvania between 2015 and 2018 who were identified as children with confirmed maltreatment or sibling on a CWS case initiated in the month of one of the children’s births. We restricted the analytic cohort to infants with PSE as determined by established claims-based methods described later.^9,10^ To support sibling fixed-effects analyses, we further limited the sample to infants with PSE who had at least 1 sibling born to the same biological mother and who were also identified with PSE. The discordant sibling sample included children who differed in their CWS involvement status at birth. In the sibling fixed-effects design, infants with PSE involved with CWS at birth were compared with their biological siblings also identified with PSE but not involved with CWS at birth.

Maternal and infant Medicaid claims were used to determine PSE based on maternal diagnoses or treatment for substance use during pregnancy or at delivery (in the month of birth or preceding 9 months) and infant diagnoses of neonatal withdrawal syndrome or PSE.^9,10^ eTable 1 in Supplement 1 presents full codes and indicators.

### Measures

#### Exposure

The primary exposure was CWS involvement during the birth month, defined by the date of CWS report. Birth month served as a proxy for involvement at delivery due to administrative data constraints. The unexposed group included infants who were not reported, were screened out, or not substantiated following a report.

#### Outcomes

Primary health and safety outcomes included (1) any injury, (2) any serious injury, (3) moderate- to high-complexity ED visits, and (4) repeat moderate- to high-complexity ED visits. Injuries were identified via *International Classification of Diseases, Ninth Revision*, and *International Classification of Diseases, Tenth Revision, Clinical Modification* codes (eTable 2 in Supplement 1). Serious injury reflects an Abbreviated Injury Scale score of 3 or higher (serious),^36^ an unclassified injury that could result in serious impairment or death (such as poisoning), or CWS-documented fatalities or near fatalities.^37^ Moderate- to high-complexity ED visits were defined by *Current Procedural Terminology *evaluation and management codes 99283-99285 and critical care codes 99291-99292.^38,39^ Birth-month encounters were excluded to ensure that the CWS involvement at birth (exposure) was not due to the same injury or ED event. For repeat ED visits, outpatient claims overlapping with an ongoing inpatient stay were excluded to avoid counting multiple services from 1 hospitalization.

To evaluate the potential for health care avoidance following CWS involvement, we examined several secondary outcomes. Preventive care included any well-child visit and on-time adherence (completion of all recommended first-year visits).^32^ Low-resource-intensity ED visits were those without hospitalization, laboratory tests, imaging, procedures, or medications.^40^ Maternal postpartum care was defined as any Medicaid claim with postpartum-related codes within 12 weeks of delivery.^41,42^

#### Covariates

Child-level covariates included sex, year of birth, birth order, low birth weight (<2500 g), and the presence of any PSE-related diagnosis on Medicaid records. CWS-documented race and ethnicity (collected from CWS administrative records as Hispanic, non-Hispanic Black, non-Hispanic White, and other [including Asian, Pacific Islander, and multiracial individuals]) were included to control for systemic disparities in child welfare reporting and health service utilization. Maternal characteristics during the perinatal period include SUD diagnosis type (none, alcohol/cannabis only, any opioid, or other/polysubstance) and receipt of medication-assisted treatment (MAT).^2^ These covariates were included because prior research has shown associations between demographic and perinatal characteristics, CWS reporting, and infant health outcomes.^3,12,13,43^

### Statistical Analysis

We first summarized demographic and health characteristics by CWS involvement at birth for both the full infants with PSE sample and the discordant sibling subsample. We used χ^2^ tests to assess differences between infants involved at birth and those who were not, within each analytic sample. We then present descriptive statistics on outcomes and describe the distributions of injury characteristics and ED visit reasons.

Our main analysis estimated a series of linear probability models (LPMs) to examine whether CWS involvement at birth was associated with our outcomes of interest. LPMs were selected to provide a common marginal-effect scale (percentage-point [pp] changes) that remains directly comparable across outcomes and specifications. We present 3 models: (1) bivariate associations, (2) adjusted for child characteristics, and (3) also adjusted for maternal SUD type and receipt of MAT during the prenatal period. Then, to account for unobserved maternal or familial factors that may confound findings, we estimated sibling fixed-effects models, restricting the sample to discordant siblings to satisfy the requirement for within-family variation in exposure (CWS involvement).^44^ Families where all siblings shared the same involvement status were excluded from these models as they do not contribute to the fixed-effects estimation. These models compare outcomes within families, thereby controlling for all stable maternal and family-level characteristics and reducing confounding due to genetic or environmental factors. The same covariates were included as in the pooled analyses, except for race and ethnicity, which vary minimally within sibling groups. Although time-variant factors could compromise inference validity, omitted confounders would likely bias estimates toward more adverse outcomes among infants involved with CWS, as involvement is more common for children with greater underlying risk and vulnerability.^45^ In addition, to test the robustness of our findings on health and safety outcomes, we assessed whether results were driven by the subset of infants entering foster care at birth by excluding these sibling groups from the models. Statistical analyses were performed using Stata, version 18.5 (StataCorp).

## Results

In this study in 5858 (3001 male and 2839 female) infants with PSE and 2782 (1388 male and 1386 female) discordant infants with PSE siblings, fewer than half of infants in the infants with PSE cohort (2418 [41.3%]) and the discordant sibling subsample (1359 [48.9%]) were involved with CWS at birth. Infants with PSE reported at birth differed significantly from those who were not reported on several characteristics (Table 1). In the full sample, involved infants were more likely to be non-Hispanic Black (719 [29.7%] compared with 795 [23.1%]) and less likely to be non-Hispanic White (1382 [57.2%] compared with 2145 [62.4%]). They were also more often born in later cohort years (eg, 2018: 858 [35.5%] vs 796 [23.1%]). Infants with PSE involved with CWS had higher birth order (1136 [50.0%] vs 1192 [34.7%] for second-born) and were more likely to have a documented PSE-related diagnosis (811 [33.5%] vs 902 [26.2%]). Maternal opioid SUD type and receipt of MAT were more common among infants not involved with CWS (49.7% vs 43.2%; MAT: 32.8% vs 27.8%). In the discordant sibling sample, differences by race and ethnicity, PSE-related diagnosis, and maternal MAT receipt were nonsignificant, but differences by birth year, birth order, and maternal SUD type remained.

**Table 1.** Characteristics of Infants With PSE, by CWS Involvement at Birth and Analytic Sample^a^

Variables^b^	Infants, No. (%)			
	All infants with PSE (n = 5858)^c^		Discordant infants with PSE siblings (n = 2782)^d^	
	Involved	Not involved	Involved	Not involved
Total	2418 (41.3)	3440 (58.7)	1359 (48.9)	1423 (51.2)
Sex				
Female	1187 (49.1)	1652 (48.0)	680 (50.0)	706 (49.6)
Male	1126 (50.7)	1775 (51.6)	679 (50.0)	709 (49.8)
Race and ethnicity^e^				
Hispanic	212 (8.8)	332 (9.7)	128 (9.4)	146 (10.3)
Non-Hispanic Black	719 (29.7)^f^	795 (23.1)	375 (27.6)	390 (27.4)
Non-Hispanic White	1382 (57.2)	2145 (62.4)^f^	787 (57.9)	822 (57.8)
Other	105 (4.3)	168 (4.9)	69 (5.1)	65 (4.6)
Year of birth				
2015	397 (16.4)	947 (27.5)^f^	200 (14.7)	414 (29.1)^f^
2016	505 (20.9)	894 (26.0)^f^	267 (19.7)	370 (26.0)^f^
2017	658 (27.2)^g^	803 (23.3)	370 (27.2)^f^	321 (22.6)
2018	858 (35.5)^f^	796 (23.1)	522 (38.4)^f^	318 (22.4)
Birth order				
1	1121 (46.4)	2072 (60.2)^f^	566 (41.7)	928 (65.2)^f^
2	1136 (50.0)^f^	1192 (34.7)	675 (49.7)^f^	408 (28.7)
≥3	161 (6.7)^b^	176 (5.1)	118 (8.7)^b^	87 (6.1)
Birth weight, g				
≥2500	2008 (83.0)	2901 (84.3)	1180 (86.8)	1222 (85.9)
<2500	410 (17.0)	539 (15.7)	179 (13.2)	201 (14.1)
PSE-related diagnosis				
No	1607 (66.5)	2538 (73.8)	942 (69.3)	1000 (70.3)
Yes	811 (33.5)^f^	902 (26.2)	417 (30.7)	423 (29.7)
Maternal SUD type				
Alcohol/cannabis only	413 (17.1)	828 (24.1)^f^	250 (18.4)	338 (23.8)^f^
Opioid (any)	1044 (43.2)	1708 (49.7)^f^	637 (46.9)	702 (49.3)
Other/polysubstance	961 (39.7)^f^	904 (26.3)	472 (34.7)^f^	383 (26.9)
Maternal MAT receipt				
No	1745 (72.2)	2312 (67.2)	931 (68.5)	952 (66.9)
Yes	673 (27.8)	1128 (32.8)^f^	428 (31.5)	471 (33.1)

Abbreviations: CWS, child welfare system; MAT, medication-assisted treatment; PSE, prenatal substance exposure; SUD, substance use disorder.

^a^ Percentages may not total 100% because of rounding or missing/unknown values.

^b^ Significant difference between infants involved vs not involved at birth within each analytic sample, *P* < .05.

^c^ The all infants with PSE group includes all infants identified with PSE who have at least 1 sibling in the dataset.

^d^ The discordant infants with PSE siblings group is restricted to families in which siblings differ in CWS involvement status at birth (ie, at least 1 infant was involved and at least 1 was not).

^e^ Race and ethnicity were collected from CWS administrative records. The other category includes infants identified in CWS administrative records as Asian, American Indian or Alaska Native, or Other Pacific Islander, or multiracial, as these groups were too small for individual statistical analysis in the current sample.

^f^ Significant difference between infants involved vs not involved reported at birth within each analytic sample, *P* < .001.

^g^ Significant difference between infants involved vs not involved reported at birth within each analytic sample, *P* < .01.

By age 1 year, 848 of 5858 infants with PSE (14.5%) had at least 1 injury claim, 492 experienced a serious injury (8.4%), 1979 had at least 1 moderate- to high-complexity ED visit (33.8%), and 840 had a repeat ED visit (14.3%) (Table 2). Among the 848 infants with PSE with an injury, the head and neck were the most commonly affected body regions (371 [43.8%]). Additionally, 20.6% involved unspecified injuries, and 7.5% were documented as fatal or near-fatal child maltreatment. The most common reasons for moderate- to high-complexity ED visits included respiratory conditions (1052/1979 [53.2%]), infections (533/1979 [26.9%]), and gastrointestinal tract/dehydration conditions (241/1979 [12.2%]).

**Table 2.** Descriptive Statistics of Infant Outcomes by Analytic Sample

Outcome^a^	Infants, No./total No. (%)	
	All infants with PSE (n = 5858)	Discordant infants with PSE siblings (n = 2782)
Any injury visits^b^	848/5858 (14.5)	390/2782 (14.0)
Most common injured body region^c^		
Head/neck	371/848 (43.8)	162/390 (41.5)
General/multiple body regions	194/848 (22.9)	97/390 (24.9)
Extremities	181/848 (21.3)	83/390 (21.3)
Any serious or above injury visits	492/5858 (8.4)	200/2782 (7.2)
Unspecified (eg, poisoning)	175/848 (20.6)	89/390 (22.8)
Fatality/near fatality		
Fatality	42/848 (5.0)	22/390 (5.6)
Near fatality	21/848 (2.5)	11/390 (2.8)
Any moderate- to high-complexity ED visits	1979/5858 (33.8)	899/2782 (32.3)
Most common visiting reasons^d^		
Respiratory conditions	1052/1979 (53.2)	487/899 (37.2)
Infections	533/1979 (26.9)	254/899 (28.3)
Serious symptoms	241/1979 (12.2)	114/899 (12.7)
Gastrointestinal tract and dehydration	241/1979 (12.2)	110/899 (12.2)
Injury and abuse	235l/1979 (11.9)	112/899 (12.5)
Repeat moderate- to high-complexity ED visits	840/5858 (14.3)	396/2783 (14.2)
Health care avoidance indicators		
Low-resource intensity ED visits	2412/5858 (41.2)	1066/2782 (38.3)
Any well-child visits	4710/5858 (80.4)	2133/2782 (76.7)
On-time well-child visits	3067/5858 (52.4)	1379/2782 (49.6)
Maternal postpartum care visits	3823/5858 (65.3)	1829/2782 (65.7)

Abbreviations: CWS, child welfare system; ED, emergency department; *ICD*, *International Classification of Diseases*; PSE, prenatal substance exposure.

^a^ Percentages for any injury, ED visits, and repeat ED visits are calculated using the full analytic sample (n = 5858 for All infants with PSE; n = 2782 for Discordant infants with PSE siblings). Percentages for detailed outcome characteristics (eg, body region of injury or reason for ED visit) are calculated among infants who experienced the corresponding event.

^b^ Any infant with an injury-related diagnosis code in any care setting (inpatient, ED, or outpatient), combined with CWS records of fatalities or near fatalities.

^c^ Each row for body-region injuries represents the percentage of children with any injury to that body region; children may be counted in multiple rows if they had injuries to more than 1 region.

^d^ Binary indicators were created for each category at the child level. Because an individual child may have multiple ED visits for different visiting reasons, these category rates are not mutually exclusive and will not sum to 100%. Detailed list of frequent *ICD* codes within each clinical category is in eTable 3 in Supplement 1.

In LPMs with the full infants with PSE cohort, CWS involvement at birth was associated with a lower probability of injury, serious injury, moderate- to high-complexity ED, and repeat ED visits during the first year of life (Figure, A; eTables 4-7 in Supplement 1). In adjusted sibling fixed-effects models, CWS involvement was associated with a lower probability of any moderate- to high-complexity ED visit (−7.3 pp; 95% CI, −10.8 to −3.9 pp) and repeat ED visits (−3.7 pp; 95% CI, −6.3 to −1.2 pp). These correspond to a 20% (95% CI, 11.0%-30.3%) reduction in any ED visit and a 24% (95% CI, 7.6%-40.1%) reduction in repeat ED visits relative to the control group mean. Results remained consistent after excluding infants who entered foster care at birth (moderate- to high–complexity ED visits, −8.3 pp; 95% CI, −12.0 to −4.6 pp; repeat moderate- to high–complexity ED visits, −4.4 pp; 95% CI, −7.2 to −1.6 pp) (eTables 8 and 9 in Supplement 1). The finding with injury outcomes was no longer statistically significant in the sibling comparison, but similar in magnitude to the full sample point estimate.

**Figure.**
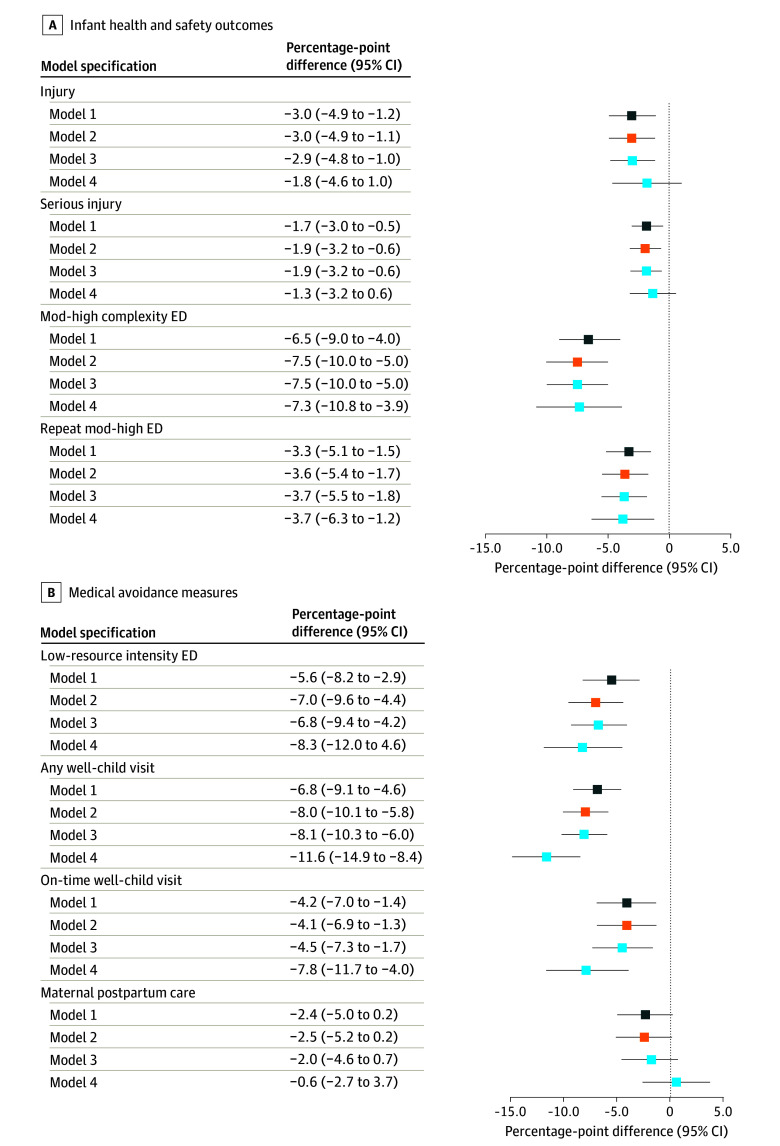
Plotted Points Graph Showing Risk Differences for CWS Involvement at Birth and Infant Outcomes Infant health and safety outcomes include any injury, serious injury, moderate- to high-complexity ED visits, and repeat moderate- to high-complexity ED visits. Medical avoidance measures include any low-resource-intensity ED visits, any well-child visits, on-time well-child visits, and maternal postpartum care visits. Estimates represent percentage-point differences from linear probability models. Point estimates are shown with 95% CIs. Model 1 displays bivariate associations; model 2 adjusted for child characteristics; model 3 additionally adjusted for maternal substance use disorder type and medication-assisted treatment; and model 4 additionally included sibling fixed effects. CWS indicates child welfare system; ED, emergency department; mod, moderate.

Potential medical avoidance is shown in the Figure, B (eTables 10-13 in Supplement 1). CWS involvement at birth was associated with lower rates of low-resource-intensity ED (fully adjusted model with sibling fixed effects, −8.3 pp; 95% CI, −12.0 to −4.6 pp) and well-child visits in sibling fixed-effects models (eg, on-time in fully adjusted model with sibling fixed effects, −7.8 pp; 95% CI, −11.7 to −4.0 pp), corresponding to 20% and 15% reductions relative to the control mean. There was no significant difference in maternal postpartum care (−0.6 pp; 95% CI, −2.7 to −3.7 pp).

## Discussion

Despite federal requirements for infants with PSE notifications, substantial variability persists in diagnosis and reporting practices, and little is known about how early CWS involvement influences infant health and safety outcomes for infants with PSE. This study addressed that gap by leveraging linked CWS and Medicaid records to examine associations between CWS involvement at birth and subsequent risks of injury and emergency care during the first year of life. Using sibling fixed-effects models to reduce family-level confounding, findings suggest that CWS involvement at birth is associated with lower rates of moderate- to high-complexity ED visits and repeat visits; estimates for child injuries followed similar patterns but were not statistically significant in the final models.

We found an association between CWS involvement at birth and reduced use of moderate- to high-complexity emergency care among infants with PSE. Although the study was not designed to identify mechanisms, sensitivity analyses allowed us to examine some possible explanations. One possibility is that less emergency care corresponds to removal from parental care. However, results were similar after excluding infants who entered foster care, indicating that the pattern also applied to infants remaining in their birth households.

A second possibility is that reduced ED use reflects health care avoidance following CWS involvement, potentially to reduce surveillance and prevent new reports. Prior research has reported similar patterns, noting that state-level policies mandating PSE-related CWS referrals are associated with declines in medical use, including prenatal and postpartum maternal care.^46^ Our findings were mixed. Involvement at birth was not associated with reductions in maternal postpartum care, despite prior evidence that such care is sensitive to CWS reporting policies.^41^ CWS involvement was associated with lower rates of any and on-time well-child visits, although reductions in preventive care were smaller than reductions in emergency care. Whether this reflects deliberate avoidance of medical care or other barriers to maintaining preventive care among households involved with CWS remains unclear.

Although well-child adherence is generally low among infants with PSE,^47,48^ formal CWS involvement (particularly when services are provided or children are placed out of home) has been linked to increased adherence.^32^ In our sample, most infants with PSE involved with CWS at birth did not receive a formal case opening and few were removed from their homes, suggesting that many mothers may experience the stigma or fear associated with CWS involvement, without receiving services that would increase capacity. However, missed well-child visits may also be related to other factors apart from avoidance, such as perceptions that the child is healthy^49^ or receipt of preventive care (eg, vaccinations) at a recent sick-child visit.^50^ In sum, lower moderate- to high-complexity ED use may reflect both improved child health and safety alongside possible health care avoidance. Additional research is needed to identify mechanisms, including increased early intervention service use^32^ or maternal substance use treatment.^2^

### Strengths and Limitations

A key strength of this study is the use of linked child welfare and Medicaid administrative data to examine the intersection of system involvement and infant health or safety outcomes. Nevertheless, several limitations remain. First, our focus was on formal CWS involvement at birth among Medicaid-enrolled mothers and children in a single state. Medicaid covers more than 40% of US births (35% in PA)^51^ and more than 75% of births to women with perinatal SUD,^52^ making this a relevant population. However, our reliance on medical claims and CWS indicators likely under-identifies infants with PSE, potentially selecting for a more clinically or socially complex sample. Caution is needed when generalizing to populations not enrolled in Medicaid, families who never have contact with CWS, or other states. Second, limitations of causal inference remain. We relied on Medicaid claims for biological mothers and their children, without paternal health data or information on shared paternity. In addition, there may be unobserved variation between siblings or time-varying maternal characteristics that affect both CWS involvement and health care use. Third, outcomes were limited to events generating a Medicaid claim, excluding injuries for which medical care was not obtained. Finally, our design cannot specify mechanisms by which CWS involvement reduces ED use.

## Conclusions

This study suggests that, despite recognition of the risks faced by infants with PSE, empirical evidence on the potential benefits and harms of early CWS involvement remains limited. This study rigorously evaluated the association between CWS involvement at birth and subsequent infant health and safety outcomes among a high-risk cohort of children identified with PSE. Using a sibling fixed-effects design, we found that CWS involvement at birth was associated with lower use of ED services in the first year of life. These results suggest that early CWS involvement may function as a critical entry point for monitoring and connecting families to services that mitigate child health risks in the first year of life. At the same time, the findings raise concerns about possible medical avoidance among families following CWS contact, suggesting the need for strategies that address child safety concerns without reducing the receipt of important preventive care services.

## Notes

Supplement 1. **eTable 1.** Indicators and data sources used to identify prenatal substance exposure **eTable 2.** ICD codes used to identify injury claims **eTable 3.** Frequent ICD codes for moderate-to-high complexity ED visits by category **eTable 4.** Association between CWS involvement at birth and injury visits **eTable 5.** Association between CWS involvement at birth and serious injury visits **eTable 6.** Association between CWS involvement at birth and moderate/high complexity ED visits **eTable 7.** Association between CWS involvement at birth and repeat moderate/high complexity ED visits **eTable 8.** Association between CWS involvement at birth and injury outcomes (excluding infants removed at birth) **eTable 9.** Association between CWS involvement at birth and ED outcomes (excluding infants removed at birth) **eTable 10.** Association between CWS involvement at birth and low-resource intensity ED visits **eTable 11.** Association between CWS involvement at birth and well-child visits **eTable 12.** Association between CWS involvement at birth and on-time well-child visits **eTable 13.** Association between CWS involvement at birth and maternal postpartum care visits

Supplement 2.
Data Sharing Statement
