# Supplementary material for: Child Welfare Involvement and Health Outcomes in Infants With Prenatal Substance Exposure
Source: JAMA Health Forum. 2026 Jun 5;7(6):e261302. doi: 10.1001/jamahealthforum.2026.1302 (PMC13241949; doi:10.1001/jamahealthforum.2026.1302)
Supplement: Supplement 1. — eTable 1. Indicators and data sources used to identify prenatal substance exposure eTable 2. ICD codes used to identify injury claims eTable 3. Frequent ICD codes for moderate-to-high complexity ED visits by category eTable 4. Association between CWS involvement at birth and injury visits eTable 5. Association between CWS involvement at birth and serious injury visits eTable 6. Association between CWS involvement at birth and moderate/high complexity ED visits eTable 7. Association between CWS involvement at birth and repeat moderate/high complexity ED visits eTable 8. Association between CWS involvement at birth and injury outcomes (excluding infants removed at birth) eTable 9. Association between CWS involvement at birth and ED outcomes (excluding infants removed at birth) eTable 10. Association between CWS involvement at birth and low-resource intensity ED visits eTable 11. Association between CWS involvement at birth and well-child visits eTable 12. Association between CWS involvement at birth and on-time well-child visits eTable 13. Association between CWS involvement at birth and maternal postpartum care visits [file jamahealthforum-e261302-s001.pdf]

Supplemental Online Content

Chen W-T, Connell CM, Font SA. Child welfare involvement and health outcomes in infants with prenatal substance exposure. *JAMA Health Forum*. 2026;7(6):e261302. doi:10.1001/jamahealthforum.2026.1302

eTable 1. Indicators and data sources used to identify prenatal substance exposure

eTable 2. ICD codes used to identify injury claims

eTable 3. Frequent ICD codes for moderate-to-high complexity ED visits by category

eTable 4. Association between CWS involvement at birth and injury visits

eTable 5. Association between CWS involvement at birth and serious injury visits

eTable 6. Association between CWS involvement at birth and moderate/high complexity ED visits

eTable 7. Association between CWS involvement at birth and repeat moderate/high complexity ED visits

eTable 8. Association between CWS involvement at birth and injury outcomes (excluding infants removed at birth)

eTable 9. Association between CWS involvement at birth and ED outcomes (excluding infants removed at birth)

eTable 10. Association between CWS involvement at birth and low-resource intensity ED visits

eTable 11. Association between CWS involvement at birth and well-child visits

eTable 12. Association between CWS involvement at birth and on-time well-child visits

eTable 13. Association between CWS involvement at birth and maternal postpartum care visits

This supplemental material has been provided by the authors to give readers additional information about their work.

© 2026 Chen W-T et al. *JAMA Health Forum*.

eTable 1. Indicators and data sources used to identify prenatal substance exposure

Indicator	Source
CWS allegation of substance-affected infant	Child Welfare Information System report record with an allegation of "substance-affected infant"
CWS allegation of caregiver substance use at birth	Child Welfare Information System report record with an allegation of "caregiver substance use" during the month of the child's birth
Foster care removal reason	Adoption and Foster Care Analysis and Reporting System (AFCARS) record indicating removal due to "parent drug use" or "parent alcohol use"
Child Medicaid: withdrawal symptoms	ICD-10: P96.1<br>ICD-9: 779.5
Child Medicaid: other prenatal substance exposure	ICD-10: P04.14 P04.16 P04.17 P04.41A P04.43 P04.44 P04.81 Q86.0<br>ICD-9: 76.70 76.71 76.72 76.73 76.75 292.0
Maternal Medicaid: substance use complicating pregnancy	ICD-10: O99.31 O99.32<br>ICD-9: 648.3
Maternal Medicaid in prenatal period: overdose	ICD-10: T4.0X1-T4.6X5 T42.0X1-T42.9X5 T43.0X1-T43.9X5 T5.9X1-T5.9X5<br>ICD-9: 965.00-965.09 969.70-969.79 98.0
Maternal Medicaid in prenatal period: opioid medication-assisted treatment (MAT)	ICD-9: 304.0 304.7 305.5<br>ICD-10: F11-F19 AND: CPT or E/M: 99205 99212-99215 H0033 H0020 G9012<br>**Or Provider Specialty**: Methadone Clinic
Maternal Medicaid in prenatal period: substance use disorder	ICD-10: F11-F16 F18 F19<br>ICD-9: 303-305
Maternal Medicaid in prenatal period: substance use service, consultation, or positive toxicology screen	ICD-10: Z71.41 Z71.51 R82.6 R78.0-R78.6<br>ICD-9: V65.42 79.93 79.99 791.9<br>**Or Provider Specialty**: Drug & Alcohol Service

© 2026 Chen W-T et al. *JAMA Health Forum*.

eTable 2: ICD codes used to identify injury claims

Category	ICD-9 Codes	ICD-10-CM Codes
Mechanical trauma through the R package *icdpicr*	800-929, 940-959	S00-S99, T07, T14, T20-T32
Unspecified harm-related		
Drug/alcohol poisoning	965.0, 970, 980	T40, T51
Other poisoning	960-964, 965.1, 965.4-965.9, 966-969, 971-979, 981-989	T36-T39, T41-T50, T52-T65
Foreign bodies	930-939	T15-T19
Asphyxiation and drowning	994.7, 994.1	T71, T75.1
Heat exposure	992	T67
Cold exposure	991.0-991.3, 991.6	T33, T34, T68
Deprivation or exposure to environmental conditions	965.0, 970, 980	T40, T51

© 2026 Chen W-T et al. *JAMA Health Forum*.

eTable 3: Frequent ICD codes for moderate-to-high complexity ED visits by category

Category	Top 5 ICD-9/10 Codes
Respiratory	J06.9, J21.9, J21.0, R05, J18.9
Infections	B34.9, H66.91, H66.92, H66.93, A08.4
Serious symptoms	R50.9, R68.13, R56.9, R68.12, R21
Gastrointestinal & dehydration	R11.10, K59.00, K52.9, R19.7, K21.9
Injury & abuse	S09.90XA, S00.83XA, T76.12XA, S00.03XA, S02.0XXA

© 2026 Chen W-T et al. *JAMA Health Forum*.

eTable 4: Association between CWS involvement at birth and injury visits

	**Full sample (n = 5,858)**			**Sibling fixed effects (n = 2,782)**		
Variables	(1) Crude	(2) + Child Characteristics	(3) + Maternal Characteristics	(1) Crude	(2) + Child Characteristics	(3) + Maternal Characteristics
CWS involvement at birth	-.030** (-.049, -.012)	-.030** (-.049, -.011)	-.029** (-.048, -.010)	-.025 (-.050, .001)	-.015 (-.042, .013)	-.018 (-.046, .010)
Sex (Male)		.015 (-.003, .033)	.015 (-.003, .033)		.010 (-.028, .049)	.011 (-.028, .049)
Race/Ethnicity (ref = NH white)						
Hispanic		-.002 (-.037, .033)	-.003 (-.038, .033)		-	-
NH black		-.024* (-.045, -.002)	-.025* (-.048, -.001)		-	-
Others		-.016 (-.062, .029)	-.017 (-.062, .029)		-	-
Year of birth (ref =2015)						
2016		-.016 (-.044, .012)	-.015 (-.044, .013)		.023 (-.041, .087)	.022 (-.042, .086)
2017		-.029 (-.059, .001)	-.029 (-.059, .001)		-.009 (-.097, .078)	-.008 (-.095, .079)
2018		-.030 (-.061, .002)	-.029 (-.061, .002)		-.006 (-.121, .109)	-.005 (-.120, .109)
Birth order (ref = 1)						
2		.022 (-.001, .044)	.022 (-.001, .044)		-.025 (-.094, .045)	-.026 (-.096, .044)
3+		.013 (-.029, .056)	.013 (-.029, .056)		-.068 (-.185, .050)	-.068 (-.186, .050)
Low birthweight		.051*** (.023, .079)	.051*** (.023, .079)		.050 (-.000, .101)	.048 (-.002, .099)
PSE diagnosis on Medicaid		.022 (-.001, .044)	.023* (.000, .045)		.067 (.029, .106)**	.067*** (.028, .105)
Maternal SUD type (ref = alcohol/cannabis only)						
Opioid (any)			-.001 (-.030, .032)			.052 (-.041, .145)
Others/polysubstance			-.007 (-.033, .019)			.050 (-.016, .117)
Maternal MAT receipt			-.008 (-.036, .020)			-.017 (-.086, .052)
Mean	.157			.151		

*$p < .05$, ** $p < .01$, *** $p < .001$

© 2026 Chen W-T et al. *JAMA Health Forum*.

eTable 5: Association between CWS involvement at birth and serious injury visits

Variables	Full sample (n = 5,858) (1) Crude	(2) + Child Characteristics	(3) + Maternal Characteristics	Sibling fixed effects (n = 2,782) (1) Crude	(2) + Child Characteristics	(3) + Maternal Characteristics
CWS involvement at birth	-.017** (-.030, -.005)	-.019** (-.032, -.006)	-.019** (-.032, -.006)	-.014 (-.031, .003)	-.012 (-.030, .007)	-.013 (-.032, .006)
Sex (Male)		.005 (-.008, .017)	.005 (-.008, .017)		.003 (-.024, .030)	.002 (-.025, .029)
Race/Ethnicity (ref = NH white)						
Hispanic		-.006 (-.028, .016)	-.006 (-.028, .017)		-	-
NH black		.001 (-.015, .016)	.002 (-.015, .019)		-	-
Others		-.028* (-.050, -.005)	-.027* (-.050, -.005)		-	-
Year of birth (ref =2015)						
2016		.003 (-.015, .022)	.003 (-.015, .022)		.022 (-.022, .065)	.022 (-.022, .065)
2017		-.005 (-.025, .016)	-.005 (-.025, .016)		.012 (-.047, .072)	.013 (-.046, .072)
2018		-.006 (-.027, .016)	-.006 (-.027, .016)		-.014 (-.092, .064)	-.014 (-.091, .064)
Birth order (ref = 1)						
2		.006 (-.010, .021)	.006 (-.010, .021)		.002 (-.045, .049)	.001 (-.046, .048)
3+		.025 (-.008, .058)	.025 (-.008, .058)		.004 (-.077, .086)	.003 (-.079, .085)
Low birthweight		.027** (.007, .047)	.027** (.007, .047)		.034 (-.003, .071)	.033 (-.003, .070)
PSE diagnosis on Medicaid		.013 (-.002, .028)	.012 (-.003, .027)		.035** (.010, .059)	.034** (.009, .059)
Maternal SUD type (ref = alcohol/cannabis only)						
Opioid (any)			.002 (-.018, .023)			-.005 (-.060, .050)
Others/polysubstance			.003 (-.015, .021)			.013 (-.027, .053)
Maternal MAT receipt			.002 (-.017, .021)			.013 (-.037, .064)
Mean	.059			.054		

*$p < .05$, ** $p < .01$, *** $p < .001$

© 2026 Chen W-T et al. *JAMA Health Forum*.

eTable 6: Association between CWS involvement at birth and moderate/high complexity ED visits

	Full sample (n = 5,858)			Sibling fixed effects (n = 2,782)		
Variables	(1) Crude	(2) + Child Characteristics	(3) + Maternal Characteristics	(1) Crude	(2) + Child Characteristics	(3) + Maternal Characteristics
CWS involvement at birth	-.065*** (-.090, -.040)	-.075*** (-.100, -.050)	-.075*** (-.100, -.050)	-.080*** (-.113, -.046)	-.070*** (-.104, -.036)	-.073*** (-.108, -.039)
Sex (Male)		.024 (-.001, .048)	.024 (-.000, .048)		.015 (-.035, .065)	.016 (-.035, .066)
Race/Ethnicity (ref = NH white)						
Hispanic		.107*** (.061, .153)	.100*** (.053, .146)		-	-
NH black		.075*** (.044, .107)	.060*** (.026, .094)		-	-
Others		.028 (-.033, .088)	.023 (-.038, .084)		-	-
Year of birth (ref =2015)						
2016		.035 (-.002, .072)	.038* (-.001, .076)		.148*** (.064, .232)	.152*** (.068, .236)
2017		-.008 (-.048, .032)	-.006 (-.046, .034)		.093 (-.016, .201)	.096 (-.012, .205)
2018		.006 (-.038, .049)	.007 (-.036, .051)		.137 (-.006, .280)	.142 (-.001, .285)
Birth order (ref = 1)						
2		.018 (-.013, .049)	.017 (-.013, .048)		-.094* (-.178, -.010)	-.094* (-.178, -.010)
3+		-.036 (-.094, .023)	-.039 (-.097, .019)		-.247*** (-.392, -.101)	-.243*** (-.388, -.098)
Low birthweight		.103*** (.067, .140)	.103*** (.066, .140)		.119*** (.049, .188)	.117*** (.047, .186)
PSE diagnosis on Medicaid		.067*** (.038, .097)	.078*** (.048, .108)		.150*** (.099, .201)	.150*** (099, .201)
Maternal SUD type (ref = alcohol/cannabis only)						
Opioid (any)			-.046* (-.090, -.003)			.036 (-.081, -.154)
Others/polysubstance			-.022 (-.059, .015)			.034 (-.049, .117)
Maternal MAT receipt			-.003 (-.040, .034)			-.075 (-.147, .332)
Mean	.365			.356		

*$p < .05$, ** $p < .01$, *** $p < .00$

© 2026 Chen W-T et al. *JAMA Health Forum*.

eTable 7: Association between CWS involvement at birth and repeat moderate/high complexity ED visits

	Full sample (n = 5,858)			Sibling fixed effects (n = 2,782)		
Variables	(1) Crude	(2) + Child Characteristics	(3) + Maternal Characteristics	(1) Crude	(2) + Child Characteristics	(3) + Maternal Characteristics
CWS involvement at birth	-.033*** (-.051, -.015)	-.036*** (-.054, -.017)	-.037*** (-.055, -.018)	-.038** (-.062, -.014)	-.034** (-.059, -.009)	-.037** (-.063, -.012)
Sex (Male)		.031*** (.014, .049)	.032*** (.014, .049)		.038* (.001, .074)	.039* (.002, .134)
Race/Ethnicity (ref = NH white)						
Hispanic		.075*** (.038, .113)	.070*** (.032, .107)		-	-
NH black		.041*** (.017, .065)	.031* (.005, .056)		-	-
Others		-.005 (-.047, .065)	-.005 (-.047, .065)		-	-
Year of birth (ref =2015)						
2016		.013 (-.014, .041)	.017 (-.011, .044)		.043 (-.017, .104)	.042 (-.018, .103)
2017		-.010 (-.040, .020)	-.008 (-.038, .022)		.033 (-.046, .111)	.034 (-.044, .112)
2018		.005 (-.027, .037)	.006 (-.026, .039)		.061 (-.042, .164)	.062 (-.042, .165)
Birth order (ref = 1)						
2		.003 (-.020, .025)	.002 (-.021, .025)		-.040 (-.101, -.021)	-.041 (-.102, -.020)
3+		-.036 (-.077, .005)	-.038 (-.080, .003)		-.100 (-.204, -.004)	-.099 (-.204, -.005)
Low birthweight		.081*** (.051, .110)	.080*** (.050, .110)		.139*** (.082, .196)	.136*** (.080, .193)
PSE diagnosis on Medicaid		.007 (-.015, .028)	.015 (-.008, .037)		.027 (-.010, .064)	.026 (-.011, .063)
Maternal SUD type (ref = alcohol/cannabis only)						
Opioid (any)			-.023 (-.055, -.010)			.079 (-.001, -.160)
Others/polysubstance			-.006 (-.035, .022)			.063* (.004, .121)
Maternal MAT receipt			-.009 (-.035, .017)			-.030 (-.094, .034)
Mean	.158			.157		

p* < .05, ** *p* < .01, * *p* < .001

© 2026 Chen W-T et al. *JAMA Health Forum*.

eTable 8: Association between CWS involvement at birth and injury outcomes (excluding infants removed at birth)

	Injury		Serious injury	
Variables	Full (n = 5,444)	FE (n = 2,496)	Full (n = 5,444)	FE (n = 2,496)
CWS involvement at birth	-.028** (-.048, -.018)	-.018 (-.046, .010)	-.021** (-.034, -.009)	-.016 (-.035, .003)
Sex (Male)	.015* (.001, .038)	.011 (-.028, .049)	.003 (-.009, .015)	.014 (-.015, .043)
Race/Ethnicity (ref = NH white)				
Hispanic	-.003 (-.041, .033)	-	-.001 (-.023, .023)	-
NH black	-.025* (-.050, -.001)	-	.001 (-.015, .017)	-
Others	-.017 (-.062, .029)	-	-.025* (-.047, -.005)	-
Year of birth (ref =2015)				
2016	-.015 (-.044, .013)	.022 (-.042, .086)	.008 (-.010, .027)	.026 (-.019, .071)
2017	-.029 (-.059, .001)	-.008 (-.095, .079)	-.001 (-.021, .018)	-.002 (-.062, .058)
2018	-.029 (-.061, .002)	-.005 (-.120, .109)	-.002 (-.022, .018)	-.012 (-.091, .067)
Birth order (ref = 1)				
2	.022 (-.001, .044)	-.026 (-.096, .044)	.006 (-.009, .021)	.017 (-.032, .065)
3+	.013 (-.029, .056)	-.068 (-.186, .050)	.020 (-.012, .052)	.023 (-.062, .109)
Low birthweight	.051*** (.023, .079)	.048 (-.002, .099)	.025* (.005, .045)	.028 (-.009, .065)
PSE diagnosis on Medicaid	.023* (.000, .045)	.067*** (.028, .105)	.013 (-.002, .028)	.024 (-.001, .049)
Maternal SUD type (ref = alcohol /cannabis only)				
Opioid (any)	-.001 (-.030, .032)	.052 (-.041, .145)	.007 (-.014, .023)	-.083 (-.071, .055)
Others/polysubstance	-.007 (-.033, .019)	.050 (-.016, .117)	.003 (-.014, .021)	.017 (-.026, .060)
Maternal MAT receipt	-.008 (-.036, .020)	-.017 (-.086, .052)	.001 (-.019, .020)	.017 (-.039, .073)
Mean	.158	.150	.059	.054

FE, fixed-effects

*p < .05, ** p < .01, *** p < .001

© 2026 Chen W-T et al. *JAMA Health Forum*.

eTable 9: Association between CWS involvement at birth and ED outcomes (excluding infants removed at birth)

	Moderate/high complexity ED visits		Repeat moderate/high complexity ED visits	
Variables	Full (n = 5,444)	FE (n = 2,496)	Full (n = 5,444)	FE (n = 2,496)
CWS involvement at birth	-.078*** (-.104, -.051)	-.083*** (-.120, -.046)	-.038*** (-.057, -.019)	-.044** (-.072, -.016)
Sex (Male)	.029 (.004, .055)	.044 (-.009, .097)	.033*** (.015, .051)	.049* (.010, .087)
Race/Ethnicity (ref = NH white)				
Hispanic	.103*** (.054, .152)	-	.070** (.031, .109)	-
NH black	.056** (.020, .091)	-	.030* (.002, .057)	-
Others	.023 (-.040, .086)	-	-.015 (-.057, .028)	-
Year of birth (ref =2015)				
2016	.039* (.001, .077)	.144** (.055, .233)	.018 (-.011, .047)	.045 (-.020, .109)
2017	-.011 (-.053, .031)	.093 (-.022, .208)	-.012 (-.043, .019)	.038 (-.045, .121)
2018	.005 (-.040, .050)	.149 (-.002, .300)	.009 (-.024, .043)	.079 (-.032, .190)
Birth order (ref = 1)				
2	.016 (-.016, .047)	-.093* (-.182, -.004)	-.002 (-.024, .023)	-.055 (-.120, .009)
3+	-.036 (-.097, .025)	-.264** (-.417 -.111)	-.042 (-.085, .002)	-.118 (-.228, -.008)
Low birthweight	.103*** (.064, .141)	.105** (.034, .176)	.083*** (.051, .113)	.132*** (.074, .190)
PSE diagnosis on Medicaid	.080*** (.048, .112)	.147*** (092, .202)	.015 (-.009, .038)	.041* (.001, .080)
Maternal SUD type (ref = alcohol /cannabis only)				
Opioid (any)	-.044 (-.089, .001)	.046 (-.082, -.174)	-.024 (-.058, -.010)	.105* (.020, .190)
Others/polysubstance	-.020 (-.058, .019)	.027 (-.060, .115)	-.006 (-.036, .024)	.062* (.002, .122)
Maternal MAT receipt	-.011 (-.050, .027)	-.064 (-.169, .412)	-.012 (-.039, .016)	-.022 (-.089, .044)
Mean	.366	.361	.159	.162

FE, fixed-effects

*$p < .05$, ** $p < .01$, *** $p < .001$

© 2026 Chen W-T et al. *JAMA Health Forum*.

eTable 10: Association between CWS involvement at birth and low-resource intensity ED visits

	Full sample (n = 5,858)			Sibling fixed effects (n = 2,782)		
Variables	(1) Crude	(2) + Child Characteristics	(3) + Maternal SUD Type	(1) Crude	(2) + Child Characteristics	(3) + Maternal SUD Type
CWS involvement at birth	-.056*** (-.082, -.029)	-.070*** (-.096, -.044)	-.068*** (-.094, -.042)	-.086*** (-.121, -.051)	-.080*** (-.116, -.043)	-.083*** (-.120, .046)
Sex (Male)		.034** (.009, .059)	.035** (.010, .060)		.021 (-.020, .073)	.022 (-.030, .074)
Race/Ethnicity (ref = NH white)						
Hispanic		.090** (.044, .137)	.080** (.033, .126)		-	-
NH black		.049** (.138, .083)	.049** (.138, .083)		-	-
Others		.033 (-.028, .094)	.027 (-.036, .088)		-	-
Year of birth (ref =2015)						
2016		.031 (-.007, .069)	.036 (-.002, .074)		.113* (.027, .198)	.116*** (.030, .201)
2017		.024 (-.018, .066)	.027 (-.016, .068)		.080 (-.039, .199)	.083 (-.036, .202)
2018		.024 (-.022, .069)	.025 (-.021, .070)		.091 (-.063, .243)	.095 (-.059, .247)
Birth order (ref = 1)						
2		.002 (-.030, .034)	-.103 (-.167, .039)		-.058 (-.151, .035)	-.057 (-.151, .037)
3+		-.065* (-.125, -.005)	.058* (-.020, .096)		-.130 (-.286, .026)	-.123 (.286, .029)
Low birthweight		.176*** (.138, .213)	.176*** (.028, .088)		.218*** (.149, .289)	.217*** (.147, .288)
PSE diagnosis on Medicaid		.094*** (.064, .125)	.111*** (.080, .143)		.166*** (.112, .219)	.166*** (113, 220)
Maternal SUD type (ref = alcohol/cannabis only)						
Opioid (any)			-.084*** (-.129, -.040)			.021 (-.105, .145)
Others/polysubstance			-.053** (-.091, -.015)			.032 (-.054, .117)
Maternal MAT receipt			-.001 (-.039, .039)			-.045 (-.144, .056)
Mean	.437			.422		

p* < .05, ** *p* < .01, * *p* < .001

© 2026 Chen W-T et al. *JAMA Health Forum*.

eTable 11: Association between CWS involvement at birth and well-child visits

	Full sample (n = 5,858)			Sibling fixed effects (n = 2,782)		
Variables	(1) Crude	(2) + Child Characteristics	(3) + Maternal SUD Type	(1) Crude	(2) + Child Characteristics	(3) + Maternal SUD Type
CWS involvement at birth	-.068*** (-.091, -.046)	-.080*** (-.101, -.058)	-.081*** (-.103, -.060)	-.134*** (-.168, -.100)	-.118*** (-.150, -.086)	-.116*** (-.149, -.084)
Sex (Male)		.015 (-.004, .033)	.016 (-.003, .034)		.023 (-.018, .063)	.022 (-.018, .063)
Race/Ethnicity (ref = NH white)						
Hispanic		.013 (-.021, .048)	-.006 (-.040, .029)		-	-
NH black		-.013 (-.037, .012)	-.050** (-.076, -.024)		-	-
Others		-.072** (-.122, -.022)	-.083** (-.133, -.034)		-	-
Year of birth (ref =2015)						
2016		.024 (-.005, .054)	.034* (.004, .064)		.158*** (.089, .227)	.160*** (.091, .229)
2017		-.062*** (-.094, -.031)	-.057*** (-.087, -.026)		.033 (-.063, .128)	.033 (-.063, .128)
2018		-.108*** (-.142, -.074)	-.104*** (-.137, -.070)		.023 (-.102, .148)	.023 (-.101, .149)
Birth order (ref = 1)						
2		.069*** (.050, .094)	.067*** (.041, .093)		-.043 (-.121, .037)	-.041 (-.120, .038)
3+		.026 (-.021, .074)	.018 (-.029, .065)		-.133 (-.267, .002)	-.132 (-.266, .028)
Low birthweight		.171*** (.154, .188)	.169*** (.152, .186)		.252*** (201, .303)	.253*** (202, .304)
PSE diagnosis on Medicaid		.239*** (.221, .257)	.267*** (.247, .286)		.417*** (.376, .459)	.418*** (.377, .459)
Maternal SUD type (ref = alcohol/cannabis only)						
Opioid (any)			-.098** (-.130, -.064)			-.052 (-.144, .040)
Others/polysubstance			-.040** (-.069, -.012)			-.038 (-.105, .028)
Maternal MAT receipt			-.021 (-.049, .008)			-.003 (-.073, .067)
Mean	.832			.827		

p* < .05, ** *p* < .01, * *p* < .001

© 2026 Chen W-T et al. *JAMA Health Forum*.

eTable 12: Association between CWS involvement at birth and on-time well-child visits

Variables	Full sample (n = 5,858)			Sibling fixed effects (n = 2,782)		
	(1) Crude	(2) + Child Characteristics	(3) + Maternal SUD Type	(1) Crude	(2) + Child Characteristics	(3) + Maternal SUD Type
CWS involvement at birth	-.042** (-.070, -.014)	-.041** (-.069, -.013)	-.045** (-.073, -.017)	-.091*** (-.129, -.052)	-.073*** (-.111, -.035)	-.078*** (-.117, -.040)
Sex (Male)		.010 (-.015, .035)	.011 (-.015, .036)		.020 (-.035, .074)	.020 (-.035, .074)
Race/Ethnicity (ref = NH white)						
Hispanic		-.040 (-.088, .009)	-.052* (-.102, -.003)		-	-
NH black		-.084*** (-.117, -.051)	-.106*** (-.142, -.071)		-	-
Others		-.056 (-.118, .006)	-.064* (-.126, -.001)		-	-
Year of birth (ref =2015)						
2016		.028 (-.011, .067)	.035 (-.004, .075)		.221*** (.131, .311)	.221*** (.131, .311)
2017		-.005 (-.046, .036)	-.001 (-.042, .041)		.183** (.059, .307)	.185** (.062, .309)
2018		-.015 (-.060, .031)	-.011 (-.056, .035)		.209* (.050, .369)	.212** (.053, .371)
Birth order (ref = 1)						
2		-.032 (-.065, .001)	-.033* (-.066, -.001)		-.193** (-.293, -.093)	-.195*** (-.295, -.095)
3+		-.095** (-.159, -.031)	-.101** (-.165, -.037)		-.214* (-.384, -.044)	-.216* (-.386, -.045)
Low birthweight		.090*** (.052, .128)	.088*** (.051, .126)	.	.164*** (.087, .241)	.161*** (.083, .238)
PSE diagnosis on Medicaid		.164*** (.133, .195)	.181*** (.150, .213)		.297*** (.241, .353)	.295*** (.239, .351)
Maternal SUD type (ref = alcohol/cannabis only)						
Opioid (any)			-.038 (-.084, .007)			.063 (-.058, .184)
Others/polysubstance			.006 (-.033, .044)			.087 (-.003, .171)
Maternal MAT receipt			-.023 (-.061, .015)			-.012 (-.112, .089)
Mean	.541			.536		

*p < .05, ** p < .01, *** p < .001

© 2026 Chen W-T et al. *JAMA Health Forum*.

eTable 13: Association between CWS involvement at birth and maternal postpartum care visits

	**Full sample (n = 5,858)**			**Sibling fixed effects (n = 2,782)**		
Variables	(1) Crude	(2) + Child Characteristics	(3) + Maternal SUD Type	(1) Crude	(2) + Child Characteristics	(3) + Maternal SUD Type
CWS involvement at birth	-.024 (-.050, .002)	-.025 (-.052, .002)	-.020 (-.046, .007)	-.004 (-.033, .025)	-.003 (-.029, .035)	-.006 (-.027, .037)
Sex (Male)		-.004 (-.030, .023)	-.003 (-.029, .024)		-.014 (-.064, .037)	-.013 (-.063, .037)
Race/Ethnicity (ref = NH white)						
Hispanic		-.014 (-.065, .038)	-.019 (-.070, .033)		-	-
NH black		.052** (.018, .085)	.035 (.001, .071)		-	-
Others		-.023 (-.088, .043)	-.026 (-.092, .040)		-	-
Year of birth (ref =2015)						
2016		-.049* (.090, -.008)	-.049* (.090, -.008)		.073 (-.009, .155)	.065 (-.0017, .147)
2017		.011 (-.034, .056)	.011 (-.036, .056)		.144* (.033, .255)	.138* (.028, .248)
2018		.028 (-.023, .080)	.027 (-.024, .079)		.212** (.072, .352)	.203** (.062, .344)
Birth order (ref = 1)						
2		-.060** (-.097, -.023)	-.060** (-.097, -.024)		-.175*** (-.264, -.088)	-.180*** (-.268, -.092)
3+		-.144*** (-.213, -.075)	-.147*** (-.216, -.078)		-.225** (-.382, -.068)	-.230** (-.387, -.072)
Low birthweight		-.008 (-.045, .028)	-.008 (-.045, .029)		.012 (-.052, .075)	.013 (-.050, .077)
PSE diagnosis on Medicaid		.010 (-.020, .039)	.018 (-.013, .049)		.013 (-.033, .058)	.011 (-.035, .057)
Maternal SUD type (ref = alcohol/cannabis only)						
Opioid (any)			-.097*** (-.144, -.049)			.038 (-.084, .158)
Others/ polysubstance			-.069** (-.108, -.031)			-.001 (-.084, .082)
Maternal MAT receipt			.039 (-.005, .081)			.084 (-.009, .176)
Mean	.663			.662		

$^{*}p < .05$, $^{**} p < .01$, $^{***} p < .001$

© 2026 Chen W-T et al. *JAMA Health Forum*.
