# Supplementary material for: Child Welfare Involvement and Health Outcomes in Infants With Prenatal Substance Exposure
Source: JAMA Health Forum. 2026 Jun 5;7(6):e261302. doi: 10.1001/jamahealthforum.2026.1302 (PMC13241949; doi:10.1001/jamahealthforum.2026.1302)
Supplement: Supplement 2. — Data Sharing Statement [file jamahealthforum-e261302-s002.pdf]

Data Sharing Statement

Chen. Child Welfare Involvement and Health Outcomes in Infants With Prenatal Substance Exposure. *JAMA Health Forum*. Published June 05, 2026.
doi:10.1001/jamahealthforum.2026.1302

Data
Data available: No
